# Colonization and Internalization of *Salmonella enterica* and Its Prevalence in Cucumber Plants

**DOI:** 10.3389/fmicb.2020.01135

**Published:** 2020-05-29

**Authors:** Kellie P. Burris, Otto D. Simmons, Hannah M. Webb, Lauren M. Deese, Robin Grant Moore, Lee-Ann Jaykus, Jie Zheng, Elizabeth Reed, Christina M. Ferreira, Eric W. Brown, Rebecca L. Bell

**Affiliations:** ^1^Department of Food, Bioprocessing and Nutrition Sciences, North Carolina State University, Raleigh, NC, United States; ^2^Department of Horticultural Science, North Carolina State University, Raleigh, NC, United States; ^3^Center for Food Safety and Applied Nutrition, U.S. Food and Drug Administration, College Park, MD, United States

**Keywords:** *Salmonella*, cucumber, fitness, internalization, blossom

## Abstract

Consumption of cucumbers (*Cucumis sativus* var. *sativus*) has been linked to several foodborne outbreaks involving *Salmonella enterica*. The purpose of this work was to investigate the efficiency of colonization and internalization of *S. enterica* into cucumber plants by various routes of contamination. Produce-associated outbreak strains of *Salmonella* (a cocktail of serovars Javiana, Montevideo, Newport, Poona, and Typhimurium) were introduced to three cultivars of cucumber plants (two slicing cultivars and one pickling) via blossoms (ca. 6.4 log_10_ CFU/blossom, 4.5 log_10_ CFU/blossom, or 2.5 log_10_ CFU/blossom) or soil (ca. 8.3 log_10_ CFU/root zone) and were analyzed for prevalence of *Salmonella* contamination (internal and external) and serovar predominance in fruit and stems. Of the total slicing fruit harvested from *Salmonella*-inoculated blossoms (ca. 6.4, 4.5, or 2.5 log_10_ CFU/blossom), 83.9% (47/56), 81.4% (48/59) or 71.2% (84/118) were found colonized and 67.9% (38/56), 35.6% (21/59) or 22.0% (26/118) had *Salmonella* internalized into the fruit, respectively. *S.* Poona was the most prevalent serovar isolated on or in cucumber fruits at all inoculation levels. When soil was inoculated at 1 day post-transplant (dpt), 8% (10/120) of the plants were shown to translocate *Salmonella* to the lower stem 7 days post-inoculation (dpi). Results identified blossoms as an important route by which *Salmonella* internalized at a high percentage into cucumbers, and *S.* Poona, the same strain isolated from the 2015 outbreak of cucumbers imported from Mexico, was shown to be well-adapted to the blossom niche.

## Introduction

An increase in the incidence of foodborne disease outbreaks associated with consumption of fresh fruits and vegetables has been reported in the United States (DeWaal and Bhuiya, 2007). *Salmonella enterica* causes an estimated 1.2 million illnesses annually (Scallan et al., 2011) and is the primary bacterial etiological agent responsible for produce-related outbreaks in the U.S. (Hanning et al., 2009). Four recent multistate outbreaks, occurring between 2013 and 2016, were linked to the consumption of fresh cucumbers (Centers for Disease Control Prevention, 2013; Angelo et al., 2015; Bottichio et al., 2016; Kozyreva et al., 2016).

While contamination of produce can occur during postharvest processing, research has shown that *Salmonella* has been associated with the growing environment (Islam et al., 2004a, b; Barak and Liang, 2008; Gallegos-Robles et al., 2008; Hanning et al., 2009; Patchanee et al., 2010; Gorski et al., 2011; Bell et al., 2012; Micallef et al., 2012) and plants can serve as niche environments for such enteric pathogens (Brandl et al., 2013). The manner in which produce contamination occurs in the field is largely unknown; however, there has been a surge in research examining routes for *Salmonella* to contaminate produce. During crop production, plants may become contaminated with *Salmonella* through untreated biological amended soil of animal origin or water, especially if overhead irrigation with surface water is used (Jacobsen and Bech, 2012; Olaimat and Holley, 2012; Allende and Monaghan, 2015; Li et al., 2015). Environmental surveillance studies have revealed surface water and sediment as the most common sources of *Salmonella* (Gorski et al., 2011; Bell et al., 2012; Micallef et al., 2012; Strawn et al., 2013). *Salmonella* have been shown to spread throughout the field via natural events, such as rain splash (Cevallos-Cevallos et al., 2012). Once it reaches the plant via splash, *Salmonella* can internalize through roots, leaves, stems, and flowers. *Salmonella* also has been shown to translocate within tomato plants and colonize fruit following leaf inoculation without inducing any symptomatic plant response (i.e., wilting, necrotic lesions, or other hypersensitive response) (Gu et al., 2011). While irrigation water is a likely source of *Salmonella* contamination in the production environment, inconsistent results have been observed relative to the ability of *Salmonella* to colonize the root zone and translocate to other tissues (Jablasone et al., 2004; Miles et al., 2009; Hintz et al., 2010; Zheng et al., 2013). *Salmonella* has been shown to invade root tissue and translocate to the shoots (Cooley et al., 2003; Klerks et al., 2007; Barak et al., 2011) but the interaction with plants is likely *Salmonella* serovar (Klerks et al., 2007; Zheng et al., 2013) and plant cultivar dependent (Han and Micallef, 2014).

A direct route for pathogens to the fruit is through contamination of flowers. Several studies have investigated whether blossom inoculation with plant pathogens can result in subsequent fruit and seed contamination (Lessl et al., 2007; van der Wolf and van der Zouwen, 2010; Gautam et al., 2011; van der Wolf et al., 2013; Dutta et al., 2014). For instance, Gautam et al. (2011) demonstrated colonization of cantaloupe fruits by *Erwinia tracheiphila* through blossom inoculation. Eight of nine plants inoculated with *Erwinia* through the flowers demonstrated symptoms of bacterial wilt and the fruit of three of the plants developed lesions (Gautam et al., 2011). Many angiosperm flowers contain a nectary that provides pollinators a source of energy as well as a primary habitat for microbes by offering higher humidity and a reservoir of nutrients (Aleklett et al., 2014). Flowers have also been examined as a route of enteric pathogen contamination for several produce commodities, including melons (Gautam, 2012), tomatoes (Zhuang et al., 1995; Guo et al., 2001; Shi et al., 2007; Zheng et al., 2013) and most recently cucumbers (Erickson et al., 2018). *Salmonella* can travel to the ovule and colonize new fruits when inoculated onto the pistil of a tomato or cantaloupe flower (Guo et al., 2001; Gautam, 2012).

In addition to dispersal and colonization via water, *Salmonella* have been shown to survive for long periods of time in the environment (Kumar et al., 2018). For example, *Salmonella* was shown to survive in the soil environment over a period of 40 days (Kumar et al., 2018). They are able to transition between hosts and can survive and persist under harsh desiccant and temperature conditions and exposure to UV radiation, all of which are common to the agricultural environment (reviewed in Brandl, 2006; Fatica and Schneider, 2011). Survival in this harsh environment can be associated with both plant (cultivar as well as growth stage) and microbial (serovar) attributes (Klerks et al., 2007; Zheng et al., 2013; Han and Micallef, 2014).

The purpose of this work was to investigate the efficiency of colonization and internalization of *Salmonella* into cucumber plants by routes of contamination consistent with current agricultural practices. The first objective sought to assess the uptake of *Salmonella* via the stems of cucumber plants after inoculation into the soil, simulating what might happen upon exposure to contaminated drip irrigation water. The second objective focused on determining if blossoms served as a potential pathogen entry point into the fruit, representing an overhead irrigation water contamination scenario. Three cucumber cultivars (“Puccini,” “Marketmore 76,” and “Thunder”) and five produce associated outbreak *S. enterica* serovars (Javiana, Montevideo, Newport, Poona, and Typhimurium) were included in the study to evaluate relative differences by plant cultivar or strain fitness. Quantitative data on prevalence and concentration of *S.* enterica in roots and on/in the fruit, and strain-specific predominance were also collected.

## Materials and Methods

### Bacterial Cultures

Five *S. enterica* serovars were obtained from the stock culture collection of the Division of Microbiology, Center for Food Safety and Applied Nutrition (CFSAN), U.S. Food and Drug Administration (U.S. FDA, College Park, MD, United States) and were all isolated from cucumber or other produce-associated outbreaks or environmental samples: *S.* Javiana (CFSAN 037803, cucumber environmental isolate, serogroup D); *S.* Montevideo (CFSAN 001232, clinical strain associated with tomato consumption; serogroup C1); *S.* Newport (CFSAN 037836, cucumber environmental isolate; serogroup C2); *S.* Poona (CFSAN 038692, cucumber isolate from 2015 outbreak, Mexico, serogroup G); and *S.* Typhimurium (CFSAN 014512, loose leaf lettuce isolate from 2006 outbreak, serogroup B).

### Inoculum Preparation

Stock cultures were stored in tryptic soy broth (TSB) (Difco, Becton, Dickinson and Company, Sparks, MD, United States) containing 25% glycerol (Acros Organics Fisher Scientific, Fair Lawn, NJ, United States) at -80°C. Cultures were plated onto tryptic soy agar (TSA) (Difco) and incubated at 37°C for 20 h. A single colony from each culture was transferred to 5 ml of TSB and incubated for 20 h at 37°C. Subsequently, each culture was harvested by centrifugation at 5,000 × g for 10 min and washed with 0.01 M phosphate-buffered saline (PBS) (pH 7.2) (Life Technologies Corporation, Grand Island, NY, United States). Washing and subsequent centrifugation were performed three times. Bacterial cultures were resuspended in 5 ml PBS, which ~9 log_10_ (CFU/ml). An equal volume of cell suspension of each serovar was combined to make up the inocula for cucumber plants. As precedent, there have been numerous studies that have used a cocktail consisting of multiple *S.* enterica serovars to identify serovar-specific responses in tomatoes (Guo et al., 2001, 2002; Zheng et al., 2013). The five-strain cocktail was further diluted in PBS to ca. 8.3 log_10_ (CFU/root zone) for the soil inoculation studies and to ca. 6.4 log_10_ (CFU/blossom), 4.5 log_10_ (CFU/blossom), and 2.5 log_10_ (CFU/blossom) for three sets of blossom experiments representing high, medium, and low levels of inoculum. Concentration of each individual serovar was confirmed to be equally distributed in the final suspension by plate count immediately before inoculation.

### Plant Preparation

Cucumber seeds (*Cucumis sativus* var. *sativus*) of cultivars “Puccini” (pickling cucumber cultivar, treated with FarMore F1400 and film coated) and “Thunder” (slicing cucumber cultivar, treated with Thiram and film coated) were purchased from Stokes Seeds Inc. (Buffalo, NY, United States) and “Marketmore 76” (slicing cucumber cultivar, untreated) was purchased from Hummert Seed Co. (St. Joseph, MO, United States). Seeds were planted in 50:50 (w:w) mix substrate [50% Sunshine Redi-Earth Professional Growing Mix (Canadian Sphagnum peat moss 50–65%, vermiculate, dolomitic lime, 0.0001% silicon dioxide) and 50% cement sand] in standard 4.5” pots and maintained in the North Carolina State University (NCSU) Phytotron greenhouse at 26°C (day) and 22°C (night) without additional artificial lighting and grown for 3 weeks (wk). Authors have the necessary authorization to carry out the experiments within the BSL-3P greenhouse with BSL-2 class organisms. Plants for use in blossom studies were then transferred to 8” pots and planted in standard mixed substrate [33% Sunshine Redi-Earth Professional Growing Mix (Canadian Sphagnum peat moss 50–65%, vermiculite, dolomitic lime, 0.0001% silicon dioxide) and 66% pea gravel (w:w)] and were placed in the NCSU Biological Safety Level-3P Phytotron greenhouse at 26°C (day) and 22°C (night) under ambient lighting. Plants used for root inoculation studies were transplanted at 20-day old to 4.5” pots containing either the 50:50 mix substrate or the standard substrate. Plants were watered twice daily, a nutrient solution (106.23 ppm N, 10.41 ppm P, 111.03 ppm K, 54.40 ppm Ca, 12.40 ppm Mg, 5.00 ppm Fe, 13.19 ppm S, 0.113 ppm Mn, 0.24 ppm B, 0.013 ppm Zn, 0.005 ppm Cu, 0.00003 ppm Co, 0.005 ppm Mo, and 11.04 ppm Na) used in the morning and reverse osmosis (RO)-purified water in the afternoon. For the first 3 weeks, plants were limited to the nutrient solution three times a wk to prevent symptoms of nutrient toxicity. Plants used for blossom inoculations were grown on trellises.

### Soil Inoculation With *S. enterica*

A total of 150 plants, ∼20 days post-planting and 1 day post-transplant (dpt), were divided into two treatment groups: a negative control group [inoculated with PBS (*n* = 30)] and an experimental group [inoculated with 4 ml of a five-strain cocktail of ca. 8 log_10_ CFU/root zone (*n* = 120)]. Plants were designated into four subgroups of two cultivars [Group 1 (control), Puccini (*n* = 15); Group 2 (treatment), Puccini (*n* = 60); Group 3 (control), Thunder (*n* = 15); and Group 4 (treatment), Thunder (*n* = 60)]. The “Marketmore 76” slicing cultivar was only used in blossom studies. Four milliliters of prepared cocktail inoculum or 4 ml PBS were directly injected into the 50:50 mix substrate at two locations. More specifically, a 10 ml serological pipet was used to make two holes (about 40-mm depth) around the rhizosphere area. Two milliliters of prepared inoculum [ca. 8.3 log (CFU/root zone)] or 2 ml PBS were applied in each hole. After absorption, holes were refilled with 50:50 mix substrate. Plants were watered in the trays to avoid splash from watering the soil surface and to ensure that no contact was made between the treatments during application and the rest of the plant to prevent cross-contamination.

### Blossom Inoculation With *S. enterica*

A total of 184 plants at the blossom stage (*n* = 50 Puccini, *n* = 42 Marketmore 76, *n* = 92 Thunder) were divided and used in three separate experimental designs. The first used cultivars “Puccini” and “Thunder” and was comprised of two treatment groups: a negative control group [inoculated with PBS (*n* = 10)] and an experimental group [inoculated with 50 μl of a five-strain cocktail of ca. 6.4 log_10_ CFU/blossom (*n* = 40)]. The second experimental design included the same two cultivars and treatment groups with lower inoculation level of ca. 4.5 log_10_ CFU/blossom. The third design using two slicer varieties, i.e., “Thunder” and “Marketmore 76” with two treatment groups: a negative control group [inoculated with PBS (*n* = 12)] and an experimental group [inoculated with 50 μl of a five-strain cocktail of ca. 2.5 log_10_ CFU/blossom (*n* = 72)]. Each experiment was replicated one additional time, for a total of two replicates. For each experiment, greater than 380 blossoms (ca. 10 blossoms/plant) were inoculated with 50 μl of the five-strain cocktail inoculum, and ~100 blossoms (ca. 5 blossoms/plant) with PBS (control).

### Recovery of Endophytically Colonized *S. enterica* From Stems

At 7 days post-inoculation (dpi), each stem from 1 cm above the 50:50 mix substrate surface was aseptically removed from a plant using scissors sterilized between cutting stems, placed into Ziploc bags for transport to the laboratory and processed and sterilized as previously described (Franz et al., 2007; Zheng et al., 2013). Plants did not remain growing after stem sacrifice. Side branches were removed aseptically, and the main stem was immediately immersed in 70% ethanol for 1 min, 5% Clorox for 1 min, 70% ethanol for 1 min, and 1% silver nitrate for 20 min. Stems were then washed in sterile deionized water for 1 min to eliminate residual silver nitrate. They were divided into 0.5 cm long pieces using a sterile scalpel in positional order from apical to basal, discarding the last basal piece. Finally, stems were sectioned and each 0.5 cm stem piece was placed immediately after sectioning onto the surface of XLD agar medium (Difco) arranged in positional order from apical to basal. Appearance of presumptively-positive *Salmonella* colonies was observed daily for up to 7 days at room temperature and stem position of presumptive positive stem pieces was recorded (Zheng et al., 2013). For those presumptively positive stem pieces, an aliquot of bacteria surrounding the stem piece was collected and re-streaked onto XLD agar with overnight incubation at 37°C for colony purification. Five colonies of differing morphology from each XLD-positive stem piece were randomly chosen for subsequent serological characterization, plated on TSA and incubated overnight at 37°C.

### Fruit Sampling and Testing Procedures

For each experiment, more than 250 cucumber fruit were harvested between 10 and 42 dpi. Fruit was carefully removed from the plant using a Ziploc bag so as not to contaminate gloves or cucumbers. Two samples were recovered from each fruit harvested (termed surface and inside). One hundred milliliters of modified Buffered Peptone Water (mBPW) (Vassiliadis et al., 1978) were added to each Ziploc bag and the cucumber was massaged for 2 min to recover *Salmonella* from the surface (Zheng et al., 2013) (termed surface samples). Following massaging, cucumber fruit was carefully removed from the Ziploc bag and placed into a 70% ethanol bath for 20 min to surface sterilize, followed by submersion in a sterile water bath for 5 min (Zheng et al., 2013). The efficacy of the surface sterilization method was previously validated in our laboratory prior to initiation of experiments (data not shown). Fruit were then dried in a laminar flow hood and aseptically cut using a standard kitchen knife into ca. 2 cm^3^ pieces. Up to ca. 200 g of cut fruit were aseptically placed into individual sterile Whirl-Pak filter bag (termed inside samples). Each bag was gently massaged and pre-enriched in 200 ml of mBPW with at least 1:1 test portion-to-broth ratio (weight to volume) at 37°C for 20–24 h. An aliquot of 0.1 ml from the incubated pre-enrichment was transferred to a culture tube containing 10 ml Rappaport-Vassiliadis medium (RV) (Rappaport et al., 1956; Vassiliadis et al., 1978) vortexed and incubated at 42°C for 20–24 h. Each incubated selective enrichment broth was streaked onto XLD agar plates (Difco) and incubated at 37°C overnight. Five colonies of differing morphology from each XLD-positive plate were randomly chosen for serological characterization, plated for purification on TSA and incubated overnight at 37°C.

### Molecular Serotyping

A DNA template was prepared from each purified isolate by resuspending a single colony into 75 μl 1× Tris-EDTA (TE) pH 8 (Thermo Fisher Scientific, Vilnius, LT, United States) and heating at 100°C for 20 min. The 20 μl PCR mixture contained HotStart MasterMix (Qiagen, Germantown, MD, United States) and 1 μl DNA template. Amplification was conducted as described by Leader et al. (2009). Briefly, a single 16-plex PCR was performed in a thermal cycler (Bio-Rad, Hercules, CA, United States) with the following parameters: initial denaturation at 94°C for 5 min, then 25 cycles of 94°C for 30 s, 57°C for 60 s, 72°C for 30 s followed by a 72°C incubation for 5 min, then 15 cycles of 94°C for 30 s, 60°C for 60 s, 72°C for 30 s followed by 72°C incubation for 5 min. The Agilent 2100 Bioanalyzer was used to analyze PCR amplicons using the DNA 1000 Labchip^®^ kit (Agilent Technologies, Santa Clara, CA, United States) (Leader et al., 2009). Unique banding patterns could distinguish each serovar from one another.

### Statistical Analysis

Data was analyzed for prevalence of contamination, serovar predominance in fruits and stems, and cultivar differences. The Pearson Chi-Square Fisher’s Exact test was used to determine significant differences in sample positivity (i.e., fruit colonization) obtained for inoculated fruit for all cultivars over all inoculum levels. For serological surveillance of *S. enterica* colonies isolated from stems, an estimated percentage was calculated for each of the serovars used in the studies. The estimated percent was calculated based on the number of isolates identified as a specific serovar divided by the total number of isolates (ca. 100 colonies per each sample type). Data for serological surveillance reflects percent colonization. This percentage was calculated as the number of cucumber fruits colonized by individual serovar, both surface and inside, divided by the total number of *Salmonella-*positive cucumbers for each inoculum level. Two-way analysis of variance (ANOVA) was performed to examine significance and interaction effects of inoculum level, cultivar, location of contamination (outer vs. inner), or serovar. The Tukey-Kramer honestly significant differences (HSD) test was used to identify significant differences in serovar fitness (*P < 0.05*). JMP (JMP Pro 13, SAS Institute Inc., Raleigh, NC, United States) software was used for all the statistical analyses.

## Results

### Internalization and Migration of *S. enterica* in Cucumber Plants via Soil

**FIGURE 1:**
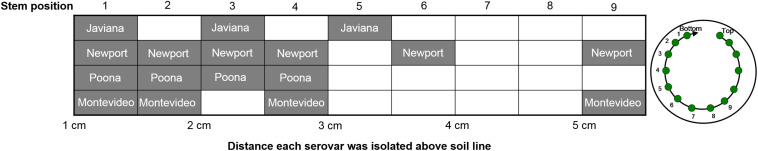
Endophytic recovery of *S. enterica* serovars and the distance traveled into the vascular stem tissue from soil inoculated with a five-strain cocktail (*S.* Javiana, Newport, Poona, Montevideo, and Typhimurium) at ca 8.3 log_10_ CFU/root zone. All stems derived from inoculated soil in the experimental group were harvested aseptically at ca. 1 cm above the soil line, sterilized, cut into ca. 0.5 cm segments and screened for endophytic populations of *Salmonella*. Molecular serotyping was used to determine the serovar of isolated colonies for each *Salmonella-*positive stem segment. Serovar Typhimurium was not identified from any of the stem segments and therefore, is not included in this figure. Stem position 1 represents ca. 1.0–1.5 cm from soil line while position 9 is closest to the apical portion of the stem (ca. 5.0–5.5 cm from soil line).

A total of 120 stems were collected at 7 dpi from plants exposed to inoculated soil, with all stems inoculated at 1 dpt. Greater than 8% (10/120) of the plants contained endophytically colonized *Salmonella* based on direct plating. Of those 10 plants found positive for *Salmonella*, 6 were cultivar Puccini (pickling cucumber) (6/60, 10%) and 4 were cultivar Thunder (slicing cucumber) (4/60, 7%). Interestingly, *Salmonella* was recovered from inside the stem ca. 5–5.5 cm above the soil line within a week following inoculation, and serovars Montevideo and Newport were recovered from stem segments farthest from the soil line (Figure 1). Of those colonies isolated from stem segments that were positive for *Salmonella* (*n* = 135), serovars Javiana, Montevideo, Newport, and Poona each were identified at 20–30%. No stem segments collected from any of the control plants (*n* = 30) were positive for the presence of *Salmonella*.

### *S. enterica* Contamination of Fruit via Blossoms

In total, 480 cucumbers [233 slicing (Table 1) and 147 pickling (Supplementary Table S1)] were harvested and analyzed for the presence of *Salmonella* on the surface and inside from blossoms inoculated with high (6.4 log_10_ CFU/blossom), medium (4.5 log_10_ CFU/blossom), and low (2.5 log_10_ CFU/blossom) inoculum concentrations. No significant differences were observed in the colonization or internalization between inoculum levels for the pickling cultivar (Supplementary Table S1). Because of this observation and the importance of slicing cucumbers associated with *Salmonella* outbreaks, slicing cultivars were focused on for prevalence of contamination analysis. Of the total slicing cucumber fruits harvested from *Salmonella*-inoculated blossoms, there was no significant difference in surface colonization by cultivar (Thunder vs. Marketmore 76; *X*^2^ = 0.935, *P* = 0.3336) or blossom inoculum level (*X*^2^ = 3.606, *P* = 0.1648; Table 1). Even at the lowest level of inoculum tested (2.5 log_10_ CFU/blossom), ca. 71% (84/118) of cucumber fruits were externally colonized with *Salmonella* (Table 1). However, significant differences were observed in internalization between inoculum levels (*X*^2^ = 34.440, *P* < 0.0001; Table 1). As levels of inoculum increased, the number of *Salmonella-*positive samples found inside the fruits increased (Table 1). However, even at the lowest level of inoculum tested, *Salmonella* was found to internalize in ca. 31% (26/84) of those cucumber fruits that were *Salmonella-*positive (Table 1).

**TABLE 1:** Prevalence of *Salmonella enterica* for slicer cultivars (Thunder or Marketmore 76) challenged via the blossom route at low (2.5 log_10_ CFU/blossom), medium (4.5 log_10_ CFU/blossom), and high (6.4 log_10_ CFU/blossom) inoculum concentrations.

Cucumber Variety and Inoculation Status	Total # of cucumbers colonized/total # of cucumbers challenged (%)*	Proportion of *S. enterica*-positive cucumbers by colonization location: **	
		# Cucumbers colonized on surface only/total # *S. enterica*- positive cucumbers (%)	# Cucumbers colonized on surface and inside***/total # *S. enterica*-positive cucumbers (%)
**HIGH INOCULUM (6.4 log_10_ CFU/BLOSSOM)**			
**Thunder**			
Inoculated blossoms^a^	47/56(83.9)a	9/47(19.1)a	38/47(80.9)a
Adjacent blossoms^b^	8/24(33.3)	7/8(87.5)	1/8(12.5)
**MEDIUM INOCULUM (4.5 log_10_ CFU/BLOSSOM)**			
**Thunder**			
Inoculated blossoms	48/59(81.4)a	27/48(56.2)b	21/48(43.8)b
Adjacent blossoms	3/32(9.4)	3/3(100.0)	0/3(0.0)
**LOW INOCULUM (2.5 LOG_10_ CFU/BLOSSOM)**			
**Thunder**			
Inoculated blossoms	48/67(71.6)a	35/48(72.9)b	13/48(27.1)c
Adjacent blossoms	1/54(1.9)	1/1(100.0)	0/1(0.0)
**Marketmore 76**			
Inoculated blossoms	36/51(70.6)a	23/36(63.9)b	13/36(36.1)c
Adjacent blossoms	0/50(0.0)	0/0(0.0)	0/0(0.0)

**^*a*^Cucumbers collected from blossoms directly challenged with the S. enterica cocktail. ^*b*^Cucumbers collected from blossoms that were in close proximity to S. enterica-inoculated blossoms. ^∗^The Pearson Chi-Square Fisher’s Exact test was used to determine significant differences in sample positivity (i.e., fruit colonization) obtained after inoculating blossoms for all cultivars over all inocula concentrations (within each column). Different letters indicate statistically significant differences (P<0.05). ^∗∗^No cucumber fruit were found contaminated inside only. ^∗∗∗^If a cucumber fruit was found positive for Salmonella inside the fruit, it also had surface contamination in every instance. External colonization with S. enterica was observed in a small number of the negative control cucumbers (5/167, 3.0%), i.e., those derived from uninoculated control plants.**

In total 160 slicing cucumbers harvested from blossoms adjacent to *Salmonella*-inoculated blossoms were analyzed for colonization and internalization (Table 1). As levels of inoculum increased, the number of *Salmonella-positive* samples obtained from fruits collected adjacent to inoculated blossoms increased significantly (*X*^2^ = 29.658, *P* < 0.0001; Table 1). However, ca. 4% (1/24) adjacent cucumber fruits were positive for internalized *Salmonella* at the highest level of inoculum tested, and no significant differences were observed in internalization between inoculum levels (*X*^2^ = 5.702, *P* > 0.05; Table 1). It is of note that we did observe external colonization of *Salmonella* in a small number of our control samples (5/167, 3%); however, none were observed to internalize *Salmonella*.

**FIGURE 2:**
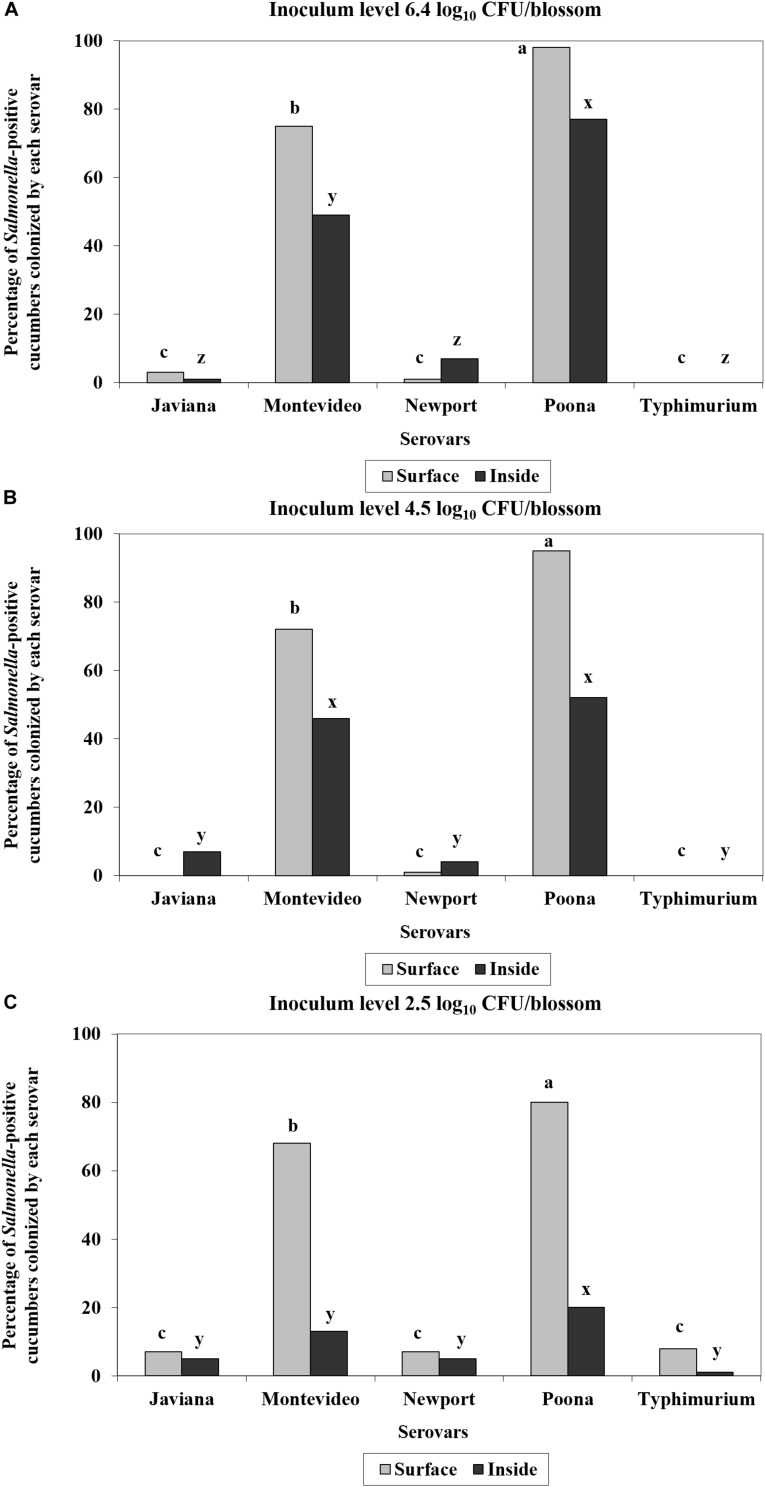
Percentage of each of the five *S. enterica* serovars identified on the surface (gray) and inside (black) of *Salmonella-*positive fruits derived from inoculated blossoms. Inoculum levels were ca. 6.4 log_10_ CFU/blossom (total number of positive fruits, *N* = 101) **(A)**; ca. 4.5 log_10_ CFU/blossom (*N* = 123) **(B)**; or ca. 2.5 log_10_ CFU/blossom (*N* = 84) **(C)**. A five-strain cocktail (*S.* Javiana, Newport, Poona, Montevideo, and Typhimurium) was inoculated onto individually labeled blossoms of cucumber plants. All the cucumber fruits derived from inoculated flowers in the experimental group were harvested and screened for surface and internal populations of *Salmonella*. Five unique colonies from each XLD-positive plate were randomly chosen for molecular serotyping. Note: multiple serovars were isolated from some of the *Salmonella*-positive cucumbers. Because there were no significant differences in predominant serotype isolated by cucumber cultivar, all data were combined for statistical analysis. Serovars with different lowercase letters (a, b, c) denote significant differences (*P*< 0.05) for surface colonization (gray), while serovars with different lowercase letters (x, y, z) denote significant differences (*P*< 0.05) for inside contamination as determined by Tukey-Kramer honestly significant difference (HSD) testing on two-way analysis of variance (ANOVA) results.

**FIGURE 3:**
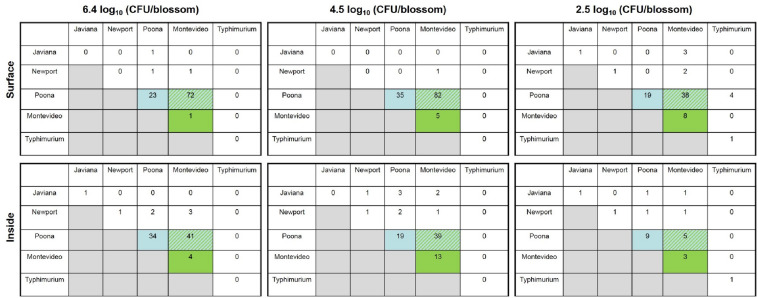
Co-colonization of each *S. enterica* serovar on both the surface (top of figure) and inside (bottom of figure) of cucumber fruit derived from blossoms inoculated with ca. 6.4 log_10_ CFU/blossom, ca. 4.5 log_10_ CFU/blossom, or ca. 2.5 log_10_ CFU/blossom. Figure represents combined data from all cultivars tested (“Thunder,” “Puccini,” and “Marketmore 76”) and all experimental replicates. A five-strain cocktail (*S*. Javiana, *S*. Newport, *S*. Poona, *S*. Montevideo, and *S*. Typhimurium) was inoculated onto individually labeled blossom of cucumber plants. All the cucumber fruits derived from inoculated flowers in the experimental group were harvested and screened for surface and internal populations of *Salmonella*. Five colonies of differing morphology from each XLD-positive plate were randomly chosen for molecular serotyping. Numbers in each box represent the number of positive cucumbers for which there is evidence of specific serovar colonization and co-colonization (*n* = 84, *n* = 123, *n* = 101 for 2.5 log_10_ CFU/blossom 4.5 log_10_ CFU/blossom and 6.4 log_10_ CFU/blossom, respectively) (light blue: *S.* Poona only; green: *S.* Montevideo only; green and blue stripe: *S.* Montevideo and *S.* Poona co-colonized). Note: triple serovars were isolated from 7 of a total of 308 *Salmonella*-positive cucumbers (*S.* Javiana, *S.* Montevideo, and *S.* Poona were isolated from five positive cucumbers – two from surface samples of fruit inoculated with 6.4 log_10_ CFU/blossom, one from surface sample of fruit inoculated with 2.5 log_10_ CFU/blossom, and two from internal samples of fruit inoculated with 4.5 log_10_ CFU/blossom; *S.* Montevideo, *S.* Newport, and *S.* Poona were isolated from one positive cucumber, the internal sample of fruit inoculated with 6.4 log_10_ CFU/blossom; *S.* Montevideo, *S.* Poona, and *S.* Typhimurium were isolated from one positive cucumber, the surface sample of fruit inoculated with 2.5 log_10_ CFU/blossom). Serovar Typhimurium was only identified from the *Salmonella-*positive cucumbers inoculated with 2.5 log_10_ CFU/blossom.

There were no significant differences in predominant serotype isolated by cucumber cultivar; therefore, pickling and slicing cultivars were combined in the subsequent analysis. *S.* Poona was the most prevalent serovar isolated from both the surface and inside of *Salmonella-*positive *c*ucumber fruits (Figure 2). Greater than 98% (99/101), 95% (117/123), and 80% (67/84) of *Salmonella-*positive cucumbers harbored *S.* Poona on the surface and greater than 77% (78/101), 52% (64/123), and 20% (17/84) were observed inside for each inoculum level, respectively (Figure 2). *S.* Montevideo was the second most prevalent serovar obtained from fruit produced from blossoms inoculated with high, medium and low inoculum levels, with greater than 75% (76/101), 72% (88/123), and 68% (57/84) found on surface for each inoculum level, respectively (Figure 2). *S.* Montevideo was also the second most prevalent serovar obtained from inside fruits produced from blossoms inoculated with high and medium inoculum levels at 49% (49/101) and 46% (57/123), respectively; however, for the lowest level of inoculum, the remaining serovars were not statistically different from each other for inside samples (Figure 2). For all three levels of inoculum, cucumbers were found to harbor more than one serovar on the surface and internally (Figure 3). *S.* Poona and *S.* Montevideo were shown to co-colonize on the surface and inside of *Salmonella-*positive cucumbers at the three inoculum levels (Figure 3). *S.* Poona was the most prevalent single serovar to colonize *Salmonella-*positive cucumbers on both the surface and inside (Figure 3).

## Discussion

Many studies have been conducted to examine the possible routes for *S. enterica* contamination of tomatoes, melons and leafy greens, but little to no work has been done on cucumbers. The goal of the present study was to evaluate the ability of *Salmonella* to colonize cucumber fruit via the inoculation of roots and blossoms during plant growth and fruit maturation. This study capitalized on access to a BSL-3P Phytotron, allowing direct work with agriculturally relevant plants and fully-virulent human outbreak strains of *Salmonella*, utilizing an increased study size as compared to growth chamber experiments and mimicking an environment relevant to field conditions.

While many studies have used relatively high concentrations of *S. enterica* to study fundamental questions about the fate of *Salmonella* in the environment, here we chose to use two different inoculation routes at different inoculum levels [ca. 8.3 log_10_ (CFU/root zone) and ca. 6.4, 4.5, and 2.5 log_10_ (CFU/blossom)] of *S. enterica* to examine its colonization and fitness in cucumber. These inoculum levels represent a range of contamination and ca. 2.5 log_10_ (CFU/blossom) is the lowest inoculum concentration examined for blossom contamination of any fruit to date. The higher inoculum levels examined would be representative of gross fecal contamination of agriculture environments in close proximity to animal operations, as naturally infected pigs may excrete *Salmonella* up to 10^6^ CFU/g feces and >5.3 log MPN/g has been found in commercial poultry house litter (Santos et al., 2005; Pires et al., 2013). However, for blossom contamination, lower inoculation levels would be anticipated in natural environments (Fravalo et al., 2003).

While screening a diversity of cucumber lineages for their responses to *Salmonella* was not the main goal of this study, it is important to note that the ability of *Salmonella* to colonize was not dependent on cucumber cultivar. Both pickling and slicer cultivars were found to colonize and internalize *Salmonella*, similarly. Each cultivar varied in its type (gynoecious, monoecious, or parthenocarpic), days from seed to fruit (50–68 days), and disease reactions. All three cultivars were resistant to scab and cucumber mosaic virus, but varied in their resistance to powdery mildew, anthracnose, angular leaf spot, and zucchini yellow mosaic virus. Therefore, we focused our data analysis on the slicer cultivar as it is most relevant to observed outbreaks and is typically consumed raw. Blossom inoculations performed in this study showed that *Salmonella* can internalize readily to fruit, pointing to its ability to evade washing. Interestingly, we observed similar rates of colonization and internalization of fruit through blossoms with all three inoculum levels (Table 1), suggesting that *Salmonella* can survive in the blossom and fruit niche at least until harvest (ca. 10–40 dpi), even when applied at ca. 2.5 log_10_ (CFU/blossom). In effect, blossoms are a direct route for pathogens to the fruit. Flowers likely offer a source of nutrients and energy as well as a hospitable habitat for microbes (Aleklett et al., 2014) and its nectary, attract pollinators which may facilitate transfer of *Salmonella* contamination from plant to plant (Aleklett et al., 2014). During reproduction, pollen grains form a tube that grows through pistil tissues to the ovule, giving a direct route of *Salmonella* internalization of the edible cucumber fruit.

At a relatively high level of inoculum [ca. 8.3 log_10_ (CFU/root zone)], we observed translocation of serovars *S.* Javiana, *S.* Newport, *S.* Poona and *S.* Montevideo to the lower stems of cucumber plants (between 3 and 5 cm above the 50:50 mix substrate line) a week following inoculation of the root zone. Previous studies examining the ability of *Salmonella* to internalize tomato plants through the root system have been conflicting (Guo et al., 2001; Miles et al., 2009; Hintz et al., 2010; Zheng et al., 2013). A multitude of factors can be responsible for these discrepancies between studies, including but not limited to: soil type, the amount of time before transplant and inoculation, cultivar used, and serotype applied. In comparison to our study, similar migration of *Salmonella* via soil has been demonstrated in tomato plants where multiple serovars (i.e., *S.* Newport, *S.* Montevideo, and *S.* Saintpaul) were recovered up to 10 cm from the soil line within a week following inoculation (Zheng et al., 2013). Our findings support a hypothesis that *Salmonella* can enter the cucumber plant through contaminated drip irrigation water and translocate up the stem, although we did not follow the plants long enough to determine the likelihood of internalization to the edible fruit. However, this method of contamination seems to be quite inefficient, at least when compared to the blossom route. While human pathogens cannot directly penetrate through root cells, previous research suggests that interior root colonization by enteric pathogens might occur passively through wounds in the roots that are damaged during transplantation or sites of lateral root emergence (Zheng et al., 2013). While *Salmonella* may be moving through the vasculature passively as one possibility, other means of active transport may exist. For example, in *Arabidopsis thaliana, Salmonella* was shown to colonize the entire plant following root inoculation and *Salmonella* was observed between epidermal cells and the vascular system (Cooley et al., 2003). The means by which *Salmonella* cells move in the vascular system needs further investigation. While many *Salmonella* serovars have been isolated from environmental samples such as surface water and sediment, only a few have been linked to multistate outbreaks associated with field and greenhouse grown cucumber – 2013 (Saintpaul), 2014 (Newport), 2015 (Poona), and 2016 (Oslo) (Centers for Disease Control Prevention, 2013; Angelo et al., 2015; Bottichio et al., 2016; Kozyreva et al., 2016). Studies that have used a cocktail of inocula containing multiple serovars of *Salmonella* have identified serovar-specific responses to tomato fruit (Guo et al., 2001; Shi et al., 2007; Zheng et al., 2013). For example, *S.* Dublin and *S.* Enteriditis were found to be less adapted to growth on or in tomato fruit than were *S.* Montevideo, *S.* Hadar, and *S.* Newport (Shi et al., 2007), and *S.* Montevideo was observed as the most prevalent serovar recovered from tomatoes following blossom inoculation (Guo et al., 2001; Shi et al., 2007; Zheng et al., 2013). Results from this study identified blossoms as an important route by which *Salmonella* internalized at a high percentage into cucumbers, and *S.* Poona, the same strain isolated from the 2015 outbreak of cucumbers imported from Mexico, was shown most adapted to the blossom niche, followed by *S.* Montevideo, another produce-outbreak associated strain. While competition between serovars can influence which species dominates within a specific plant niche (Shi et al., 2007), we found that certain outbreak-associated strains (i.e., *S.* Poona and *S.* Montevideo) appeared to be better adapted for survival and persistence in cucumber plants than were those strains of environmental origin (i.e., *S.* Newport, *S.* Javiana, and *S.* Typhimurium). Interestingly, oftentimes *S.* Poona and *S.* Montevideo co-colonized, suggesting their robust fitness in the cucumber blossom and fruit niche. Additionally, no differences were noted among serovars recovered from stem samples in terms of prevalence except *S.* Typhimurium at 7 dpi via roots. However, it appeared that *S.* Newport and *S.* Montevideo were translocated the farthest up the stem compared to other serovars tested. Of note, previous research has shown that serovars of *Salmonella* that typically associate with poultry, i.e., *S.* Enteritidis, were less adapted and fit in tomatoes than was *S.* Montevideo (Shi et al., 2007). In a recent field study by Erickson et al. (2018), directly spraying attenuated *S.* Typhimurium contaminated irrigation water onto the cucumber flower resulted in 90–100% contamination of the ovary and flower at 0 day, but only 10–40% after 3 days, indicating the relative poor fitness of *S.* Typhimurium to the cucumber blossom niche.

One potential limitation of our study was the use of a *Salmonella* cocktail (*S.* Javiana, *S.* Montevideo, *S.* Newport, *S.* Poona, and *S.* Typhimurium) for inoculations, possibly introducing culture bias during enrichment. For example, *S.* Typhimurium was not observed to colonize blossoms at the higher inoculum levels, but was observed in fruit when introduced at lower levels. While enrichment bias may potentially be a problem, no other experimental methods exist, other than emerging metagenomic technologies that may eventually prove capable of overcoming this issue. Additionally, bias may also be attributed to the limited number of colonies (five for each sample) chosen for screening, which can be resolved by bioplexing the enrichment directly; however, this would require specialized equipment. We intentionally designed our experiments to examine serovar fitness simultaneously with colonization. Because we now know that colonization occurs at low levels of inoculum and that there is selective colonization, future work is in progress to examine individual serovar colonization at the lowest levels of contamination.

Even under the most stringent control using a BSL-3P Phytotron greenhouse, we observed a small degree of *Salmonella* cross-contamination to our negative controls. In an effort to identify the reservoir of this contamination, various samples were taken within the greenhouse. In those samples, we identified plant debris located on the floor near treatment plants as a potential source of *Salmonella.* In recent research, airborne soil particulates were identified as vehicles for *Salmonella* contamination of tomatoes through blossom inoculation (Kumar et al., 2017). In that study, tomato fruits or their associated calyces, developed after exposure to *Salmonella-*contaminated airborne soil particulates distributed onto blossoms, were found positive for *S.* Newport at 67 and 78%, respectively. Interestingly, *Salmonella* was also found internalized by the tomato fruit (Kumar et al., 2017). However, cross-contamination observed in our negative controls did not result in internalization.

Even when dealing with a crop that is typically drip irrigated, the risk of exposure of blossoms to *Salmonella* via spray could occur as a consequence of frost protection measures, fertilization or fumigation/pesticide applications, or by birds or animal intrusion. Overall, the ramifications of the blossom data are substantial as specific protection of blossoms, which is part of the edible reproductive body of a seed plant, and has been included in current recommendations for best practices in the fresh produce production environment. Current studies are examining alternative barriers to prevent the spread of pathogens through wind and water from dust and manure to produce near confined-animal feedlot operations (CAFOs) (reviewed in Gutierrez-Rodriguez and Adhikari, 2018).

This work clearly demonstrates the ease with which cucumber- and outbreak-associated *Salmonella* strains colonize and internalize healthy cucumber plants and fruit, particularly through the blossom route. We observed similar rates of colonization of fruit through blossoms with all inoculum levels tested, indicating that *Salmonella* can survive in the blossom and fruit niche at least until harvest (ca. 10–40 dpi), even when applied at levels as low as ca. 2.5 log_10_ CFU/blossom. Because *S.* Poona was isolated from an outbreak associated with cucumbers, it was not surprising it was the most prevalent serovar isolated from cucumber fruit. While this study was conducted in a greenhouse, it could be expected that a similar phenomenon would occur in open production environments. In light of these data, it may be important to further consider the potential role of current field applications encompassing overhead irrigation and fumigation in the subsequent downstream contamination of mature cucumber fruits.

## Data Availability Statement

The datasets generated for this study are available on request to the corresponding author.

## Author Contributions

KB, OS, L-AJ, JZ, ER, CF, EB, and RB designed the experiments. KB, HW, LD, and RM collected the data. KB analyzed the data and wrote the manuscript draft. KB, OS, HW, LD, RM, L-AJ, JZ, ER, CF, EB, and RB edited and approved the manuscript.

## Conflict of Interest

The authors declare that this study received funding from Lifesource Biomedical LLC. The funder was not involved in the study design, collection, analysis, interpretation of data, the writing of this article or the decision to submit it for publication.
