# Supplementary material for: Colonization and Internalization of Salmonella enterica and Its Prevalence in Cucumber Plants
Source: Front Microbiol. 2020 May 29;11:1135. doi: 10.3389/fmicb.2020.01135 (PMC7273826; doi:10.3389/fmicb.2020.01135)
Supplement: Supplementary file 1 [file Table_1.docx]

**Supplemental Table 1.** Prevalence of *Salmonella enterica* contamination of cucumber fruit via blossoms from pickle cultivar (Puccini) challenged at medium (4.5 log_10_ CFU/blossom) and high (6.4 log_10_ CFU/blossom) inoculum concentrations.

|  |  | **No. of *S. enterica*-positive samples from:** | |
| --- | --- | --- | --- |
| **Group** | **# colonized/total # of cucumbers (%)** | **Surface only/total # (%)** | **Both surface and inside/total # (%)** |
| **High inoculum** |  |  |  |
| **Puccini** |  |  |  |
| **Inoculated blossoms** | 55/69 (79.7) | 6/69 (8.7) | 49/69 (71.0) |
| **Adjacent blossoms** | 3/39 (7.7) | 1/39 (2.6) | 1/39 (2.6)* |
| **Control** | 2/42 (4.8) | 1/42 (2.4) | 0/42 (0.0)* |
| **Medium inoculum** |  |  |  |
| **Puccini** |  |  |  |
| **Inoculated blossoms** | 75/78 (96.2) | 13/78 (16.7) | 62/78 (79.5) |
| **Adjacent blossoms** | 7/46 (15.2) | 6/46 (13.0) | 1/46 (2.2) |
| **Control** | 3/43 (7.0) | 2/43 (4.7) | 0/43 (0.0)* |

**Salmonella* was shown to internalize, but not colonize the surface of a single cucumber.
